# Integrated Clinical and Prognostic Analysis of the m^6^A RNA Methylation Regulator YTHDF3 in Pan-Cancer and its Correlation with Cancer Cell Proliferation

**DOI:** 10.7150/jca.78403

**Published:** 2022-11-21

**Authors:** Leiqun Cao, Bingjie Zeng, Yulan Wang, Xianzhao Wang, Yueyang Qin, Congcong Zhang, Mengyi Wu, Jiayi Wang, Xiao Zhang, Lifang Ma

**Affiliations:** 1 Anhui University of Science and Technology School of Medicine, Huainan, Anhui, 232001, China.; 2 Department of Clinical Laboratory Medicine, Shanghai Chest Hospital, School of Medicine, Shanghai Jiao Tong University, Shanghai, 200030, China.; 3 Shanghai University of Traditional Chineses Medicine, Shanghai, 201203, China.; 4 Shanghai Institute of Thoracic Oncology, Shanghai Chest Hospital, School of Medicine, Shanghai Jiao Tong University, Shanghai, 200030, China.

**Keywords:** YTHDF3, Cancer, Prognosis, Immunosuppressive microenvironment, Methylation

## Abstract

**Background:** N6-methyladenosine (m^6^A) is the most abundant and extensive chemical modification of mammalian RNA molecules. Although numerous studies have investigated m^6^A methylation-related genes, to the best of our knowledge, none have examined the expression patterns of YTH N6-methyladenosine RNA binding protein 3 (YTHDF3) across cancers.

**Methods:** Using various publicly available datasets, we searched for a potential carcinogenic role of YTHDF3 in 33 tumor types. Furthermore, the clinicopathological parameters, clinical prognostic value, enrichment analysis, mutations, microsatellite instability (MSI), tumor mutation burden (TMB), levels of infiltrating cells, and related immune checkpoint genes were included. Finally, we performed a validation analysis using existing clinical samples and proliferation-related functional experiments.

**Results:** YTHDF3 is highly expressed in most cancer types and associated with patient prognosis in certain tumors. The ROC analysis suggested that YTHDF3 has high diagnostic value in 13 types of cancer. Furthermore, we found that the genes associated with YTHDF3 were enriched for translation initiation and mRNA metabolic processes. The results of the GSEA enrichment suggest that YTHDF3 may be associated with different pathways in cells in various tumor types. We further analyzed the correlations between YTHDF3 expression and MSI, TMB, and immune checkpoint genes. YTHDF3 also possibly exerts important antitumor immunotherapy effects. Additionally, the results of the immune analysis using TIMER showed that high YTHDF3 expression levels in pan-cancer tissues were related to an immunosuppressive microenvironment. Finally, we experimentally demonstrated that both overexpression and downregulation of YTHDF3 can affect cancer cell proliferation rates.

**Conclusion:** YTHDF3 is a promising biomarker for cancer diagnosis. This study provides the first comprehensive pan-cancer report on YTHDF3 and increases our understanding of its oncogenic role in different tumors.

## Introduction

Tumorigenesis is a complex process that can involve immune evasion and cell proliferation enhancement 1, 2. Both the incidence and mortality rates of cancer are currently very high 3, 4. Thus, more effective and tolerable anticancer therapies are urgently needed. Various old and new anticancer approaches, such as surgery, radiotherapy, chemotherapy, targeted therapy, and immunotherapy, all have negative effects and obvious limitations, suggesting that combination and synergistic therapies may offer a more complete solution 5-7. With research advancements and the advent of the cancer genomics era, an increasing number of new therapeutic targets are being discovered for cancer 8, 9. Many clinical trials are underway globally to explore the clinical benefits of new combination and synergistic therapies 10, 11. However, the prognosis of cancer patients remains poor 12. To address this, epigenetic mechanisms require further investigation because an altered epigenetic state is commonly observed in all cancer types 13-15. Another key consideration is cancer immunotherapy, which can be used to manipulate a patient's own immune system to fight the cancer 16, 17. Hence, there is an urgent need to obtain a better understanding of the molecular pathogenesis of tumors in these datasets in the future. We can perform various analyses using certain public databases that contain information from different tumor genomic datasets.

Specifically, cancer cells can acquire the ability to metastasize through genetic and epigenetic changes. One such change is the modification of messenger RNA (mRNA) transcripts. N6-methyladenosine (m^6^A) is the most abundant and extensive form of internal RNA modification 18, 19. In previous studies, the regulatory role of m^6^A RNA modifications in various cellular functions, such as stem cell maintenance, cell differentiation, circadian rhythm, and neuronal function, has been revealed 20-22. YTH N6-methyladenosine RNA binding protein 3 (YTHDF3) is a member of the YTH domain family and the "reader" of m^6^A-modified mRNA 23, 24. Changes in the proteins that recognize, write, or remove m^6^A can lead to profound alterations in cellular processes and play a key role in pathological conditions, including cancer 25. The fate and function of m^6^A-methylated mRNA are primarily reader-mediated and include proteins of the YTH domain family (YTHDF1, YTHDF2, YTHDF3, and YTHDC1), other insulin-like growth factor 2 mRNA-binding proteins (IGF2BP1/2/3), and a heterogeneous nuclear ribonucleoprotein (HNRP) family (HNRNPA2B1 and HNRNPC) that can directly bind and read the m^6^A sites on mRNA molecules 26-28. The m^6^A reader YTHDC2 can reportedly bind SLC7A11 mRNA to promote lung cancer progression, while the m^6^A reader YTHDF3 binds ZEB1 mRNA to promote circ_KIAA1429, leading to hepatocellular carcinoma (HCC) progression 29, 30. However, the epigenetic mechanisms and biological functions of YTHDF3 in the pathogenesis of different cancers have been less well studied.

In this study, we comprehensively conducted bioinformatic and other related experiments involving the m^6^A reader YTHDF3. By integrating data from several databases, such as The Cancer Genome Atlas (TCGA), the Genotype-Tissue Expression (GTEx) Project, and the Cbioportal database, with network resources, such as TIMER, UALCAN, and the String website, various associations between YTHDF3 and various cancers could be presented visually. From these findings, we predicted the YTHDF3 expression levels at both the transcriptional (mRNA) and protein levels, as well as their association with clinical indicators and prognosis. Furthermore, we examined the enrichment function of YTHDF3-related genes, levels of microsatellite instability (MSI), and tumor mutation burden (TMB). The correlations between YTHDF3 and both inhibitory/stimulatory immune checkpoint genes and infiltrating immune cells were also investigated. By utilizing clinical tissue samples and conducting related functional experiments, we verified the effect of YTHDF3 in multiple cancers. We observed that YTHDF3 is widely expressed in various cancers, which may affect patient prognosis through interactions with tumor-infiltrating immune cells. These results suggest a potential scenario in which targeting YTHDF3 may be a novel therapeutic method for treating cancer.

## Materials and methods

### Data sources

The RNA sequencing (RNA-seq) data from TCGA and GTEx in the transcripts per million reads (TPM) format were uniformly processed by the Toil process using UCSC XENA (https://xenabrowser.net/datapages/). RNA-seq data in the TPM format were compared between samples after Log^2^ transformation.

### Cell culture

The human bronchial epithelial cell line (16HBE), human lung epithelial cell line (BEAS2B), human normal liver cell line (QSG-7701), human esophageal cancer cell lines (KYSR450 and TE-1), and human lung cancer cell lines (H441, H1299, H1650, H2030, A549, Calu-1, H1975, and PC9) were purchased from Fuheng Cell Center (Shanghai, China). All cells were plated in 6-well plates at a density of 100,000 cells per well in high-glucose DMEM supplemented with 1% penicillin/streptomycin (Invitrogen) and 10% fetal bovine serum (FBS; HyClone) and incubated in 5% CO_2_ at 37°C. Three-dimensional (3D) cell culture was performed by embedding cells in Cultrex® Basement Membrane Extract (BME) according to the manufacturer's instructions. The day before the experiment, the BME was prechilled at 4°C. First, 50 μL of BME and 100 μL of medium were added to each well of a 96-well plate. Subsequently, the cell suspension containing 8,000-10,000 cells was plated into each well and allowed to adhere. Then, the whole assembly was placed in a 37°C CO_2_ incubator. 3D cell growth was observed and recorded after a week. The medium was changed every three days.

### Clinical information

In total, 345 patients with non-small cell lung cancer (NSCLC), 50 patients with esophageal carcinoma, and 30 patients with thymoma were recruited from Shanghai Chest Hospital for this study, and their cancer tissue and adjacent tissue samples were obtained. The differences in YTHDF3 expression levels between cancerous and adjacent tissues were detected. The ethics and research committees of the Shanghai Chest Hospital approved the experiments.

### Inclusion and exclusion criteria

To participate in the study, each patient passed a screening evaluation according to the medical therapeutic process and physical examination. Patients were included if they met all the following inclusion criteria: (1) Age ≥ 18 years; (2) ECOG performance status of 0 or 1; (3) Histologically or cytologically proven; (4) Primary thoracic tumor; (5) Received no pre-cancer treatment other than surgery. Patients were excluded if they met any of the following exclusion criteria: (1) Presence of more than one tumor; (2) Incomplete medical records; (3) Unknown race/ethnicity; (4) Impaired organ function.

### Gene expression assays

Western blot (WB) analysis, enzyme-linked immunosorbent assay (ELISA), immunofluorescence (IF), and immunohistochemistry (IHC) were performed according to conventional protocols. The antibody against YTHDF3 (25537-1-AP, 1:1000 dilution for WB and 1:500 for IF and IHC) was purchased from Proteintech. The YTHDF3 ELISA kit was purchased from Yingxin Biotech Ltd. (Shanghai, China) to detect the YTHDF3 protein levels. IHC scores are presented by multiplying the staining intensity grade (0, 1, 2, or 3 for negative, weakly positive, moderately positive, and strong positive, respectively) and positivity rate score (0, 1, 2, 3, or 4 for ≤ 5%, 6% to 25%, 26% to 50%, 51% to 75%, and ≥ 76%, respectively).

### Cell proliferation assay

The effects of YTHDF3 knockout and overexpression on thoracic tumor cell proliferation rates were determined by MTT assays, which were conducted in 96-well plates at a density of 5 x 10^3^ cells in 100 µL culture medium per well. Medium without cells was used as a blank. After the indicated incubation times of 0, 24, 48, 72, or 96 hours, 10 μL of MTT solution was added to each well. Finally, the OD 450 values were measured to determine cell viability levels. The experiments were performed in triplicate for each group.

### Plasmid construction

YTHDF3 was knocked out in H1299 and H1975 cells by CRISPR/Cas9, and the YTHDF3 primers were annealed, digested, and connected with T4. The sgRNA was cloned into the lentiCrisprV2 plasmid (Addgene, Cambridge, MA, USA). For the CRISPR/Cas9 knockout of YTHDF3, the specific RNA sequences were sgRNAF1: 5'-CACCGGATGGTGTATTTAGTCAACC-3', sgRNAR1: 5'-AAACGGTTGACTAAATACACCATCC-3' and sgRNAF2: 5'-CACCGGCTGCAGTGACAAAAACTGT-3', sgRNAR2: 5'-AAACACAGTTTTTGTCACTGCAGCC-3'. The lentivirus-based pLVX plasmid overexpressing YTHDF3 was purchased from Zuorun Biotechnology Co., Ltd. (Shanghai, China) to generate YTHDF3-overexpressing cell lines. The protein expression of YTHDF3 in multiple transgenic cell lines was analyzed by WB.

### RNA-seq database analysis

We used the publicly available gene expression datasets from the TCGA and GTEx databases. RNA-seq data in TPM format were uniformly processed by the Toil process by UCSC XENA (https://xenabrowser.net/datapages/), then compared between samples after log2 transformation. The R package ggplot2 was used for data visualization. UALCAN (http://ualcan.path.uab.edu/analysis-prot.html) was used to perform protein expression analysis using the Clinical Proteomic Tumor Analysis Consortium (CPTAC) database, an interactive online resource for analyzing cancer omics data. We selected six different cancer types (ovarian cancer, HCC, lung adenocarcinoma (LUAD), colon cancer, uterine corpus endometrial carcinoma (UCEC), and breast cancer) from the website to compare the total YTHDF3 protein expression levels between primary tumors and normal tissues. The diagram of YTHDF3 expression in all TCGA tumors at different pathological stages and patient ages was obtained by transforming the expression data by log2 (TPM) + 1. The gene expression HTSeq - RNA-seq fragments per kilobase of exon model per million mapped fragments (FPKM) format data and clinical data of each cancer were downloaded from TCGA (https://portal.gdc.cancer.gov/). The R package ggplot2 was used to better visualize the data.

### Survival prognosis analysis

The gene expression HTSeq-RNA-seq FPKM format data and clinical data were used to examine the overall survival (OS), disease-specific survival (DSS), and progression-free interval (PFI). We adopted the log-rank test to show the results. Hazard ratios (HRs) were used as a measure of the prognostic value. HR > 1 suggests an increased risk of death. FPKM were transformed to TPM and analyzed according to the molecular expression after grouping. The R package survminer was used to better visualize the data. The R package survival was used for the statistical analysis of the survival data.

### Receiver operating characteristic (ROC) curve statistical analysis

All statistical analyses were conducted using R (v.3.6.3) software. The R package pROC was used for the ROC statistical analysis. The R package ggplot2 was used to better visualize the data. The RNA-seq data in TCGA and GTEx TPM format were uniformly processed by the Toil process by UCSC XENA (https://xenabrowser. net/datapages/).

### Genetic alteration analysis

Using the Cbioportal website (https://www.cbioportal.org/), we checked the genetic variation characteristics of YTHDF3. The results were obtained by selecting “Quickselect” and setting “YTHDF3” to “TCGA Pancancer”. In addition, GSCA (http://bioinfo.life.hust.edu.cn/GSCA/#/) is an integrated platform for genomic, pharmacogenomic, and immunogenomic gene set cancer analyses. We selected the "mutation" module to determine the impact of single nucleotide variation (SNV) and copy number variation (CNV) on survival.

### YTHDF3-related gene enrichment analysis

First, we searched the string website (https://cn.string-db.org) after entering the single protein name "YTHDF3" and the biological name "HOMO" (Homo sapiens). Then, we selected “full Network” in the parameter setting interface Network type. Next, we selected the option “active interaction sources” for all. Finally, the effectiveness of the proposed method was verified by experiments. The top 20 YTHDF3-related binding proteins were generated. We searched the GeneMANIA website (https://genemania.org/) and then entered a single protein named "YTHDF3" in the upper left corner. Different types of YTHDF3-binding proteins were produced.

To maintain consistency, the top 20 YTHDF3-related binding proteins were generated. Then, we merged the YTHDF3-related genes from the two websites. A gene ontology (GO) enrichment analysis and Kyoto Encyclopedia of Genes and Genomes (KEGG) pathway analysis were carried out using the corresponding data generated by the website analysis interface.

### Gene set enrichment analysis (GSEA)

The correlations between YTHDF3 mRNA expression levels and all genes were analyzed using R (v.3.6.3). A differential expression analysis was performed using the R package DESeq2. The R package ggplot2 was used to better visualize the data. GSEA was performed using the R package clusterProfiler. GSEA was performed using c2.cp.v7.2.symbols.gmt gene sets. A false discovery rate (FDR) < 0.25 and an adjusted *P*-value < 0.05 were considered representative of significant enrichment.

### The correlations between YTHDF3 expression and immune checkpoint (ICP) genes, TMB, and MSI

The relationships between YTHDF3 expression and the TMB, immune checkpoint genes, and MSI were searched via the SangerBox website (http://sangerbox.com/Tool), which is based on TCGA data sets.

### Immune infiltration analysis

We used an enhanced version of TIMER2.0 (http://timer.comp-genomics.org/) that integrates multiple state-of-the-art algorithms for the immune infiltration estimation. The degree of immune infiltration in YTHDF3 was evaluated by the TIMER platform (“TIDE”, “MCPCOUNTER” and “EPIC” databases) and R-ssGSEA algorithm. We used the TIMER2.0 web server "immune gene" module to explore the pan-cancer expression levels of YTHDF3 and the relationship between immune filtrates. We chose “cancer-associated fibroblasts” and then selected “submit”. The *P*-value and the partial correlation (cor) value were obtained through a simple correction of the Spearman rank correlation test. The data are visualized as heatmaps and scatter plots.

Furthermore, gene expression HTSeq - RNA-seq FPKM format data and clinical data of each cancer were downloaded from TCGA. We removed information from adjacent tissues to filter the data. The R package GSVA was used to describe the lollipop graph using R (v.3.6.3). SSGSEA was performed using the R package GSVA to quantify the infiltration levels of different immune cell types. The selected immune cells included activated dendritic cells (ADCs), B cells, CD8+ T cells, cytotoxic cells, DCs, eosinophils, immature DCs (iDCs), macrophages, mast cells, neutrophils, natural killer (NK) CD56bright cells, NK CD56dim cells, NK cells, Plasmacytoid DCs (pDCs), T cells, T helper cells, T central memory cells (Tcm), T effector memory cells (Tem), T follicular helper cells (Tfh), T gamma delta cells (Tgd), Th1 cells, Th17 cells, Th2 cells, and regulatory T cells (Tregs).

### Statistical analysis

We adopted the Kruskal-Wallis test and the Bonferroni correction significance level of multiple hypothesis testing (Dunn's test) to show the results of gene expression analysis. FPKM were transformed to TPM and analyzed after log^2^ transformation. The correlations were performed by the Spearman method. All data are presented as mean ± Standard Error of Mean (SEM). At least three independent experiments were performed and used for statistical analysis by SPSS software. The Student's t-test was performed to evaluate the difference between two groups, while differences in multiple groups of samples were analyzed using two-way analysis of variance (ANOVA). PSM was performed to minimize the impact of potential bias due to imbalanced clinicopathological parameters and accurately evaluate the correlation between YTHDF3 expression and clinical stage. Briefly, PSM analysis was conducted using a logistic regression model in which stage was used as PSM dependent variable, and age, sex and smoking status were used as PSM independent variables. Then, chi-square test was used to compare the differences and correlations between groups. The tests were regarded as statistically significant at *P < 0.05, **P < 0.01, ***P < 0.001, and ****P < 0.0001.

## Results

### YTHDF3 is highly expressed in pan-cancer data

**Figure 1:**
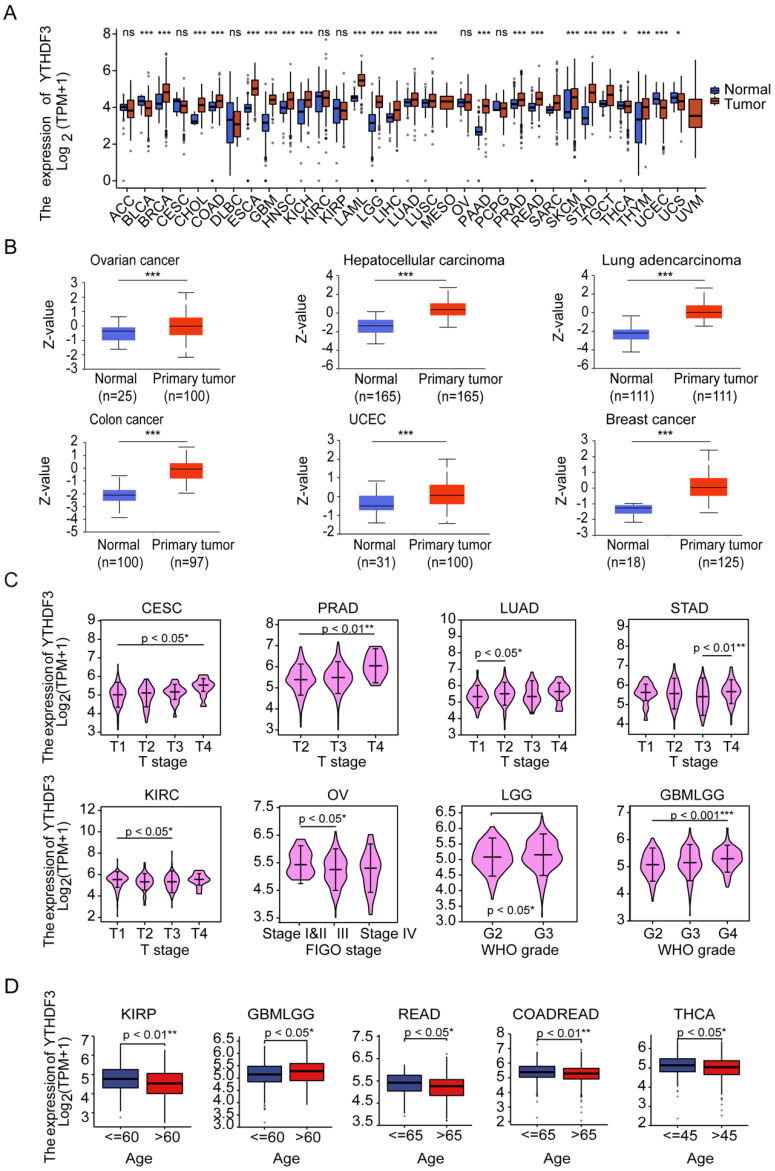
**Pan-cancer analysis of YTHDF3**. (A) YTHDF3 RNA-sequencing (RNA-seq) data in GTEx and TCGA human cancer databases. Not significant (ns), *P* ≥ 0.05; * *P* < 0.05; *** *P* < 0.001. (B) YTHDF3 protein expression levels in normal tissues and primary tumor tissues in six cancer types from the CPTAC data source. *** *P* < 0.001. (C) YTHDF3 expression levels were correlated with clinical variables stage (CESC, PRAD, LUAD, STAD, KIRC) and WHO grade (LGG, GBMLGG) and FIGO stage (OV) in TCGA database. * *P* < 0.05; ** *P* < 0.01; *** *P* < 0.001. (D) Comparisons of YTHDF3 expression levels in Clinical variables - Age (KIRP, GBMLGG, READ, COADREAD, THCA). *, *P* < 0.05; **, *P* < 0.01.

First, the expression levels of YTHDF3 in the pan-cancer data from TCGA and GTEx were evaluated. The analysis revealed that YTHDF3 expression was higher in 19 tumors, including Breast invasive carcinoma (BRCA), Cholangiocarcinoma (CHOL), Colon adenocarcinoma (COAD), Esophageal carcinoma (ESCA), Glioblastoma multiforme (GBM), Head and Neck squamous cell carcinoma (HNSC), Kidney Chromophobe (KICH), Acute Myeloid Leukemia (LAML), Lower Grade Glioma (LGG), Liver hepatocellular carcinoma (LIHC), LUAD, Lung squamous cell carcinoma (LUSC), Pancreatic adenocarcinoma (PAAD), Prostate adenocarcinoma (PRAD), Rectum adenocarcinoma (READ), Skin Cutaneous Melanoma (SKCM), Stomach adenocarcinoma (STAD), Testicular Germ Cell Tumors (TGCT), and Thymoma (THYM). In contrast, YTHDF3 expression was low in Bladder Urothelial Carcinoma (BLCA), Thyroid carcinoma (THCA), UCEC, and Uterine Carcinosarcoma (UCS) (**Figure 1A**). Additionally, the CPTAC database contains the protein expression of YTHDF3 in only relatively few cancer types. The results showed higher YTHDF3 protein expression levels in primary breast cancer, HCC, ovarian cancer, colon cancer, UCEC, and LUAD tissues compared with normal tissues (**Figure 1B**, *P* < 0.001).

We also used the "Pathological Stage Map" and "Age" modules to observe the correlation between YTHDF3 expression and cancer pathological stage, including the expression of YTHDF3 in Cervical squamous cell carcinoma and endocervical adenocarcinoma (CESC), PRAD, LGG, GBMLGG, LUAD, STAD, Ovarian serous cystadenocarcinoma (OV), and Kidney renal clear cell carcinoma (KIRC). YTHDF3 expression was significantly correlated with pathological stage in Kidney renal papillary cell carcinoma (KIRP), GBMLGG, READ, Colon adenocarcinoma/Rectum adenocarcinoma Esophageal carcinoma (COADREAD), and THCA, as well as with age (*P* < 0.05 for both **Figure 1C**, **Figure 1D**). YTHDF3 expression showed no significant correlations in the remaining cancer types (**Figure S1A**, **Figure S1B**). The results also showed that the expression levels of YTHDF3 significantly differed by the pathologic stage and age.

### YTHDF3 expression is related to prognosis in various cancer types

**Figure 2:**
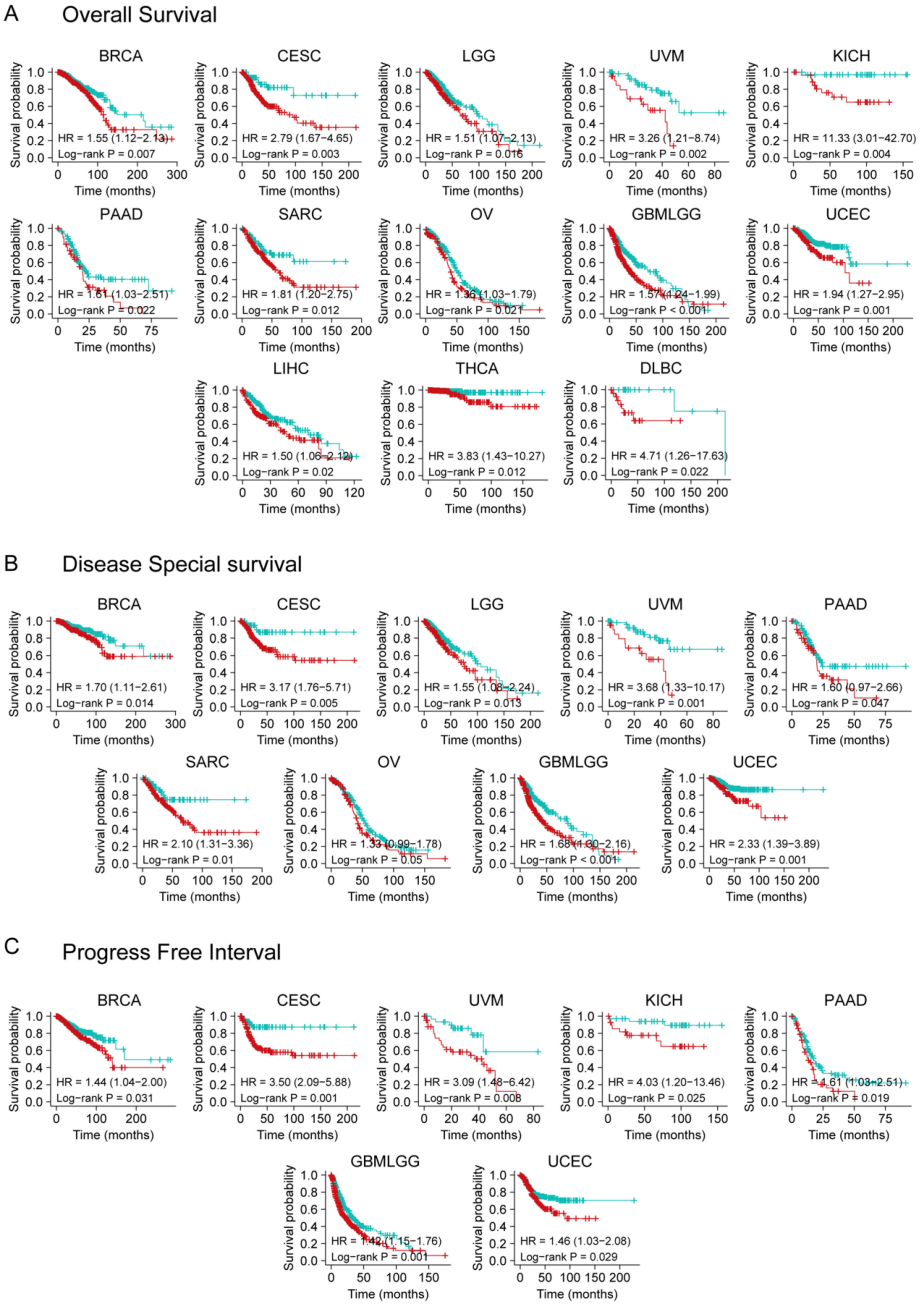
** Kaplan-Meier survival curve analysis of YTHDF3 expression levels in pan-cancer**. (A) Different expression levels of YTHDF3 in TCGA human cancer database were analyzed for overall survival (BRCA, CESC, LGG, UVM, KICH, PAAD, SARC, OV, GBMLGG, UCEC, LIHC, THCA, and DLBC). Significance is indicated by *P* < 0.05; (B) Different expression levels of YTHDF3 in TCGA were analyzed for disease-specific survival (BRCA, CESC, LGG, UVM, PAAD, SARC, OV, GBMLGG, and UCEC). Significance is indicated by *P* < 0.05; (C) Different expression levels of YTHDF3 in TCGA were analyzed for progression-free interval (BRCA, CESC, UVM, KICH, PAAD, GBMLGG, and UCEC). Significance is indicated by *P* < 0.05.

We divided cancer cases into high and low expression groups using TCGA dataset to effectively analyze different tumors. A relationship was observed between YTHDF3 expression and prognosis. As shown in **Figure 2A**, high expression of YTHDF3 was linked to poor OS in the following cancers: BRCA (HR = 1.55, *P* = 0.007), CESC (HR = 2.79, *P* = 0.003), LGG (HR = 1.51, *P* = 0.016), UVM (HR = 3.26, *P* = 0.002), KICH (HR = 11.33, *P* = 0.004), PAAD (HR = 1.61, *P* = 0.022), SARC (HR = 1.81, *P* = 0.012), OV (HR = 1.36, *P* = 0.021), GBMLGG (Glioma) (HR = 1.57, *P* < 0.001), UCEC (HR = 1.94, *P* = 0.001), LIHC (HR = 1.50, *P* = 0.02), THCA (HR = 3.83, *P* = 0.012), and Lymphoid Neoplasm Diffuse Large B-cell Lymphoma (DLBC) (HR = 4.71, *P* = 0.022) within the TCGA project. The DSS analysis data (**Figure 2B**) showed correlations between high YTHDF3 expression and poor prognosis in the TCGA cases of BRCA (HR = 1.70, *P* = 0.014), CESC (HR = 3.17, *P* = 0.005), LGG (HR = 1.55, *P* = 0.013), UVM (HR = 3.68, *P* = 0.001), PAAD (HR = 1.60, *P* = 0.047), SARC (HR = 2.10, *P* = 0.01), OV (HR = 1.33, *P* = 0.05), GBMLGG (HR = 1.68, *P* < 0.001), and UCEC (HR = 2.33, *P* = 0.001). Additionally, highly expressed YTHDF3 was linked to poor PFI in the following cancers: BRCA (HR = 1.44, *P* = 0.031), CESC (HR = 3.50, *P* = 0.001), UVM (HR = 3.09, *P* = 0.008), KICH (HR = 4.03, *P* = 0.025), PAAD (HR = 1.61, *P* = 0.019), GBMLGG (HR = 1.42, *P* = 0.001), and UCEC (HR = 1.46, *P* = 0.029) (**Figure 2C**).

We also utilized the Kaplan-Meier plotter website (KM plotter.com) to perform survival analysis for YTHDF3 as a biomarker of lung cancer, gastric cancer, breast cancer, and ovarian cancer (**Figure S2A-B**). As predicted on the Kaplan-Meier plotter website, low expression of YTHDF3 was associated with poor OS in lung cancer (*P* = 0.000018). In contrast, high expression of YTHDF3 was associated with poor post-progression survival (PPS) in lung cancer (*P* = 0.000074). Low expression of YTHDF3 was associated with poor OS (*P* = 0.000016) and PPS (*P* = 1 x 10^-8^) in gastric cancer (**Figure S2A-B**).

Our results suggest that a significant correlation exists between high YTHDF3 expression and poor prognosis across cancers. Taken together, these results demonstrate that YTHDF3 expression is closely related to prognosis in various cancer types. YTHDF3 could be a prognostic pan-cancer biomarker, and these findings deserve further investigation.

### *YTHDF3* mRNA expression has high diagnostic value in 13 cancer types

**Figure 3:**
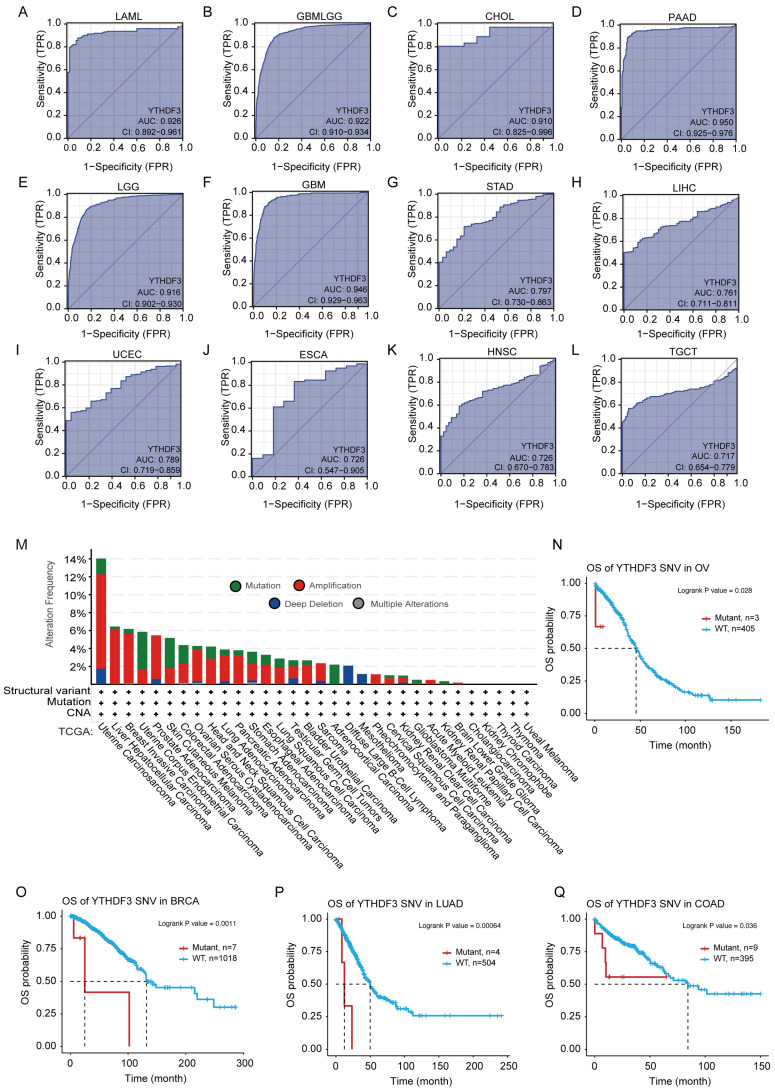
** Receiver operator characteristic curves were established to estimate the value of *YTHDF3* mRNA expression as a biomarker in pan-cancer, and YTHDF3 associations with mutations were analyzed in the TCGA data source.** (A-L: LAML, GBMLGG, CHOL, PAAD, LGG, GBM, STAD, LIHC, UCEC, ESCA, HNSC, TGCT). AUC > 0.7; (M) Expression levels of YTHDF3 gene mutations in pan-cancer were analyzed though the TCGA data source using the cBioPortal website. (N-Q) YTHDF3 SNV was linked to poor patient overall survival for BRCA, OV, LUAD, and COAD. Significance is indicated by *P* < 0.05.

To assess the diagnostic value of YTHDF3, we plotted ROC curves and calculated the AUCs for various cancers. As shown in **Figure 3A-L**, the AUC of YTHDF3 in each cancer type was 0.926 (LAML), 0.922 (GBMLGG), 0.910 (CHOL), 0.950 (PAAD), 0.916 (LGG), 0.946 (GBM), 0.797 (STAD), 0.761 (LIHC), 0.789 (UCEC), 0.726 (ESCA), 0.726 (HNSC), and 0.717 (TGCT). Our data suggest that YTHDF3 has good diagnostic value in these 12 cancer types. The remaining cancer type had a smaller AUC, and the predictive power of YTHDF3 expression was less accurate for predicting normal and tumor outcomes (AUC < 0.7) (**Figure S3A-P**). These observations warrant further exploration of the potential role of YTHDF3 in tumorigenesis. Furthermore, this information may help clinicians with cancer diagnosis and establishing treatment plans for patients.

### The "magnification" and "mutation" types of copy number alteration (CNA) occur at varying frequencies across different tumor types

We observed samples in the TCGA data set of genetically modified states in different tumors for YTHDF3. As shown in **Figure 3M**, "magnification" is the primary type of CNA in the UCSC data, with the highest frequency of change > 10%. The frequency of YTHDF3 (~4%) occurs in patients with UCS tumors, which assumes "mutation" as the main type (**Figure 3M**). Notably, all DLBC and Pheochromocytoma and Paraganglioma (PCPG) cases with genetic changes (from 1% to 2% frequency) had missing copies of YTHDF3, and all Adrenocortical carcinoma (ACC) cases with genetic changes (~2% frequency) were mutation types (**Figure 3M**).

Different from somatic gene CNAs, we searched the GSCA website for detailed information regarding diagnostic SNVs and germline gene CNVs in the YTHDF3 cohort. We generated a Kaplan-Meier curve to show the significant differences (*P* ≤ 0.05) in survival according to the details regarding diagnostic SNVs/CNVs in the GSCA platform. As shown in **Figure 3N-Q**, YTHDF3 SNV was linked to poor OS in the following cancers: BRCA (*P* = 0.0011), OV (*P* = 0.028), LUAD (*P* = 0.00064), and COAD (*P* = 0.036) from the TCGA database. Simultaneously, YTHDF3 CNV was linked to poor OS in the following cancers: UVM (*P* = 0.00016), UCEC (*P* = 0.0015), KIRC (*P* = 0.04), and KIRP (*P* = 0.00014) (**Figure S4A**). YTHDF3 CNV was linked to poor DSS in the following cancers: UVM (*P* = 0.00014), UCEC (*P* = 0.0019), KIRC (*P* = 0.0017), KIRP (*P* = 4.3 x 10^-5^), THYM (*P* = 0.0028), and THCA (*P* = 0.013) (**Figure S4B**). We did not analyze the relationships between clinical survival outcomes and YTHDF3 expression in detail because of missing clinical expression data for YTHDF3. This gene is a promising biomarker for predicting cancer patient survival, and is therefore worthy of future studies.

### YTHDF3 is involved in RNA translation

**Figure 4:**
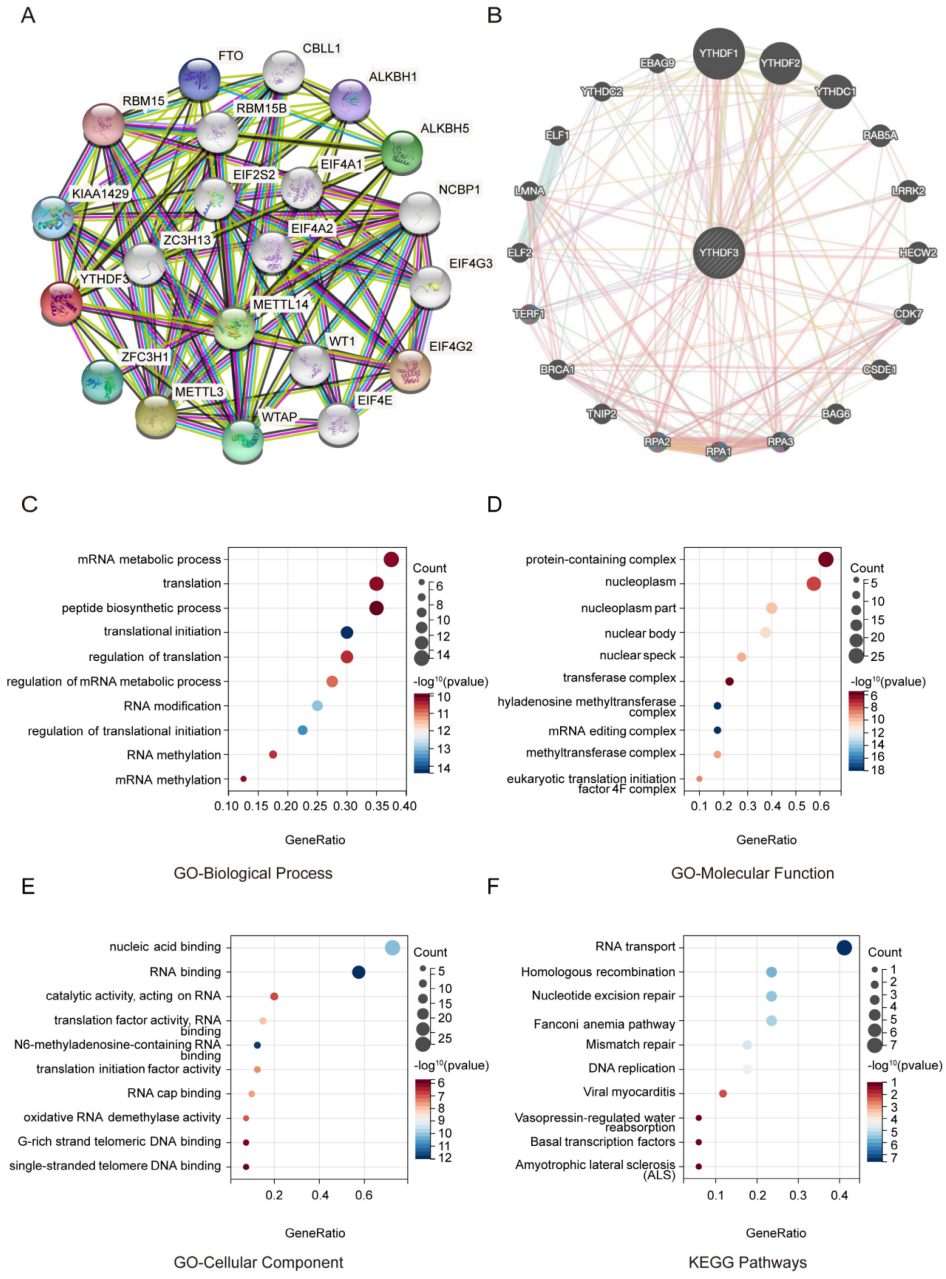
**Functional enrichment of YTHDF3-associated genes in pan-cancer.** (A) YTHDF3-associated genes are shown in a network generated with the STRING website. (B) YTHDF3-associated genes are shown in an elliptical network generated with GeneMANIA. (C) GO-Biological Process; (D) GO-Molecular Function; (E) GO-Cellular Component; (F) KEGG Pathways.

To further study the molecular mechanism of YTHDF3 in tumorigenesis, we conducted a series of pathway enrichment analyses targeting YTHDF3-binding proteins and YTHDF3 expression-related genes. Using the String Tool and GeneMANIA database, we identified 40 YTHDF3-binding proteins supported by experimental evidence (**Figure 4A-B**). We further explored the potential features. The path is based on the use of genes previously mined on the string website using the Cluster Programmer R package. The functional enrichment and GO analysis showed that YTHDF3 is mainly associated with RNA methylation and involved in the process of RNA metabolism (**Figure 4C-4E**). In addition, the KEGG pathway enrichment analysis indicated that these genes were mainly enriched in RNA transport and homologous recombination (**Figure 4F**). These results strongly suggest that YTHDF3 could be related to RNA translation and may be involved in the effect of YTHDF3 on tumor pathogenesis.

### YTHDF3 is associated with various pathways in different tumor cell types

**Figure 5:**
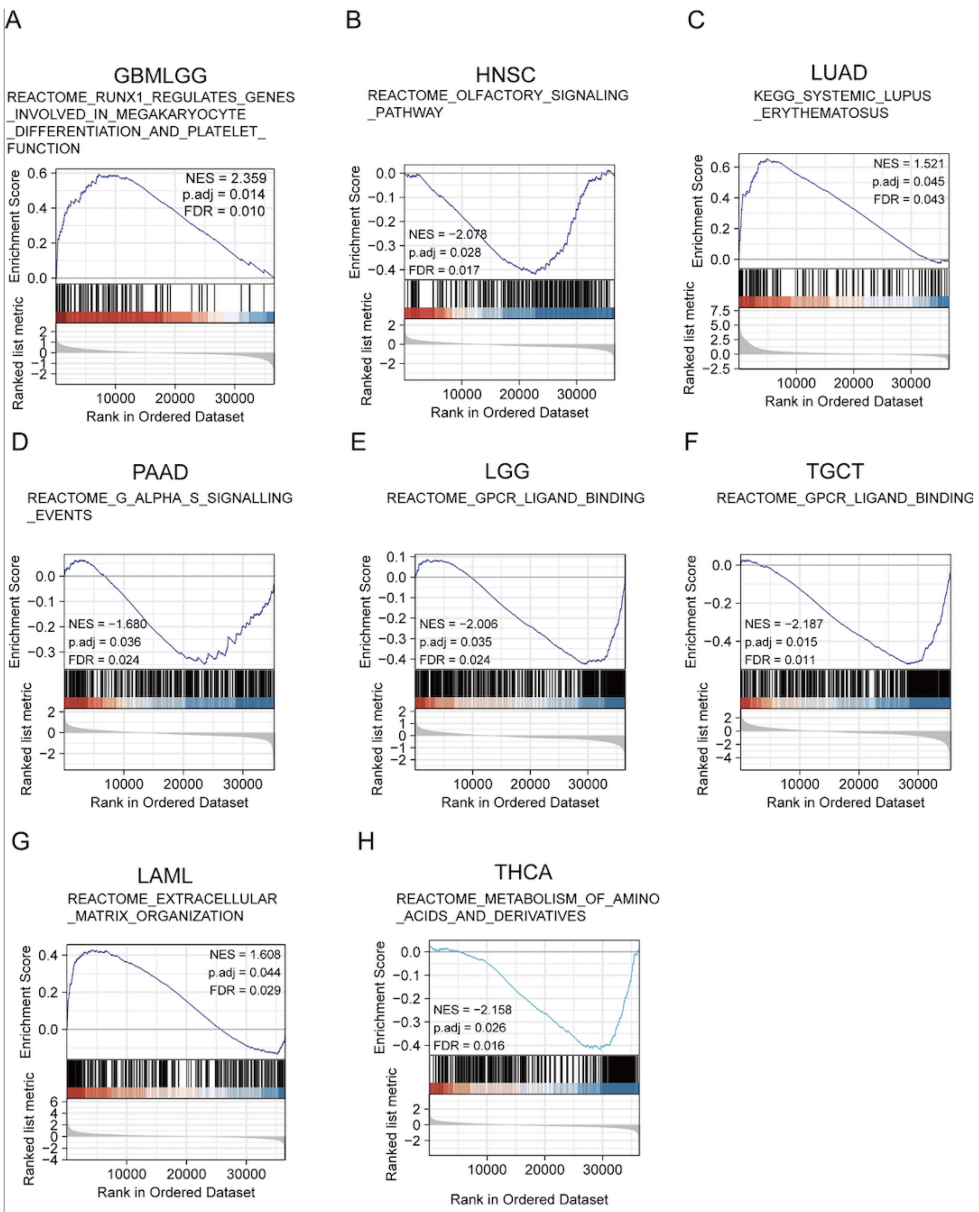
** Gene set enrichment analysis (GSEA). (A-H)** YTHDF3 gene enrichment in pan-cancer was analyzed by GSEA (GBMLGG, HNSC, LUAD, PAAD, LGG, TGCT, LAML, THCA). False discovery rate (FDR) < 0.25; significant *P* adjust. < 0.05.

To identify the molecular pathways enriched for YTHDF3, we examined activated signaling pathways across cancers by GSEA. The results showed that the upregulated pathways were involved in REACTOME_RUNX1_REGULATES_GENES_INVOLVED_IN_MEGAKARYOCYTE_DIFFERENTIATION_AND_PLATELET_FUNCTION (GBMLGG: NES = 2.35; *P* adj. =0.014; FDR = 0.010) (**Figure 5A**), KEGG_SYSTEMIC_LUPUS_ERYTHEMATOSUS (LUAD: NES = 1.521; *P* adj. = 0.045; FDR = 0.043) (**Figure 5C**), and REACTOME_EXTRACELLULAR_MATRIX_ORGANIZATION (LAML: NES = 1.608; *P* adj. = 0.044; FDR = 0.029) (**Figure 5G**), while the downregulated pathways were involved in REACTOME_OLFACTORY_SIGNALING_PATHWAY (HNSC: NES = -2.078; *P* adj. = 0.028; FDR = 0.017) (**Figure 5B**), REACTOME_G_ALPHA_S_SIGNALLING_EVENTS (PAAD: NES = -1.680; *P* adj. = 0.036; FDR = 0.024) (**Figure 5D**), REACTOME_GPCR_LIGAND_BINDING (LGG: NES = -2.006; *P* adj. = 0.035; FDR = 0.028) (**Figure 5E**), REACTOME_GPCR_LIGAND_BINDING (TGCT: NES = -2.186; *P* adj. = 0.015; FDR = 0.011) (**Figure 5F**), and REACTOME_METABOLISM_OF_AMINO_ACIDS_AND_DERIVATIVES (THCA: NES = -2.158; *P* adj. = 0.026; FDR = 0.016) (**Figure 5H**). The GSEA enrichment results for the remaining cancer types were not statistically significant. These results suggest that, in various tumor types, YTHDF3 may be associated with different pathways that impact the effects of YTHDF3 on tumor pathogenesis.

### YTHDF3 expression can affect MSI and the TMB in the pan-cancer analysis

To evaluate early immunotherapy efficacy, we further analyzed the correlations between YTHDF3 expression and MSI and TMB. This gene can possibly exert important antitumor immunotherapy effects. Our findings show that YTHDF3 expression had significant positive associations with MSI in stomach and esophageal carcinoma (STES), STAD, and CESC, while it had negative associations in DLBC, PRAD, HNSC, THCA, GBMLGG, BRCA, and LUAD (**Figure S5A**). In contrast, TMB was positively correlated with YTHDF3 expression in STAD and negatively correlated with YTHDF3 expression in THCA (**Figure S5B**). These features indicate that YTHDF3 can influence antitumor immunity by regulating mechanisms involving the immune system and composition of the tumor microenvironment (TME).

### YTHDF3 expression is related to immune checkpoint genes across cancers

Immune checkpoint genes have well-established important impacts on immunotherapy and tumor-infiltrating immune cells. To explore this, we examined the associations between immune checkpoint genes and YTHDF3 expression across cancer. We found that the expression levels of immune checkpoint inhibitory genes and immune checkpoint stimulatory genes were strongly associated with YTHDF3 expression in pan-cancers, such as UVM, DLBC, OV, KIPAN, PAAD, KIRC, BRCA, and SARC, among 70 immune checkpoint genes (**Figure S6**). Particular attention should be given to OV, in which 67 of 70 immune checkpoint genes were related to YTHDF3 expression. Expression of immune checkpoint genes was observed in all cancer types. Therefore, we concluded that YTHDF3 is a potential new target for immunotherapy drugs. This finding may help improve the accuracy of clinical decisions and predict patient response to immunotherapy.

### YTHDF3 expression is related to an immunosuppressive microenvironment across cancers

**Figure 6:**
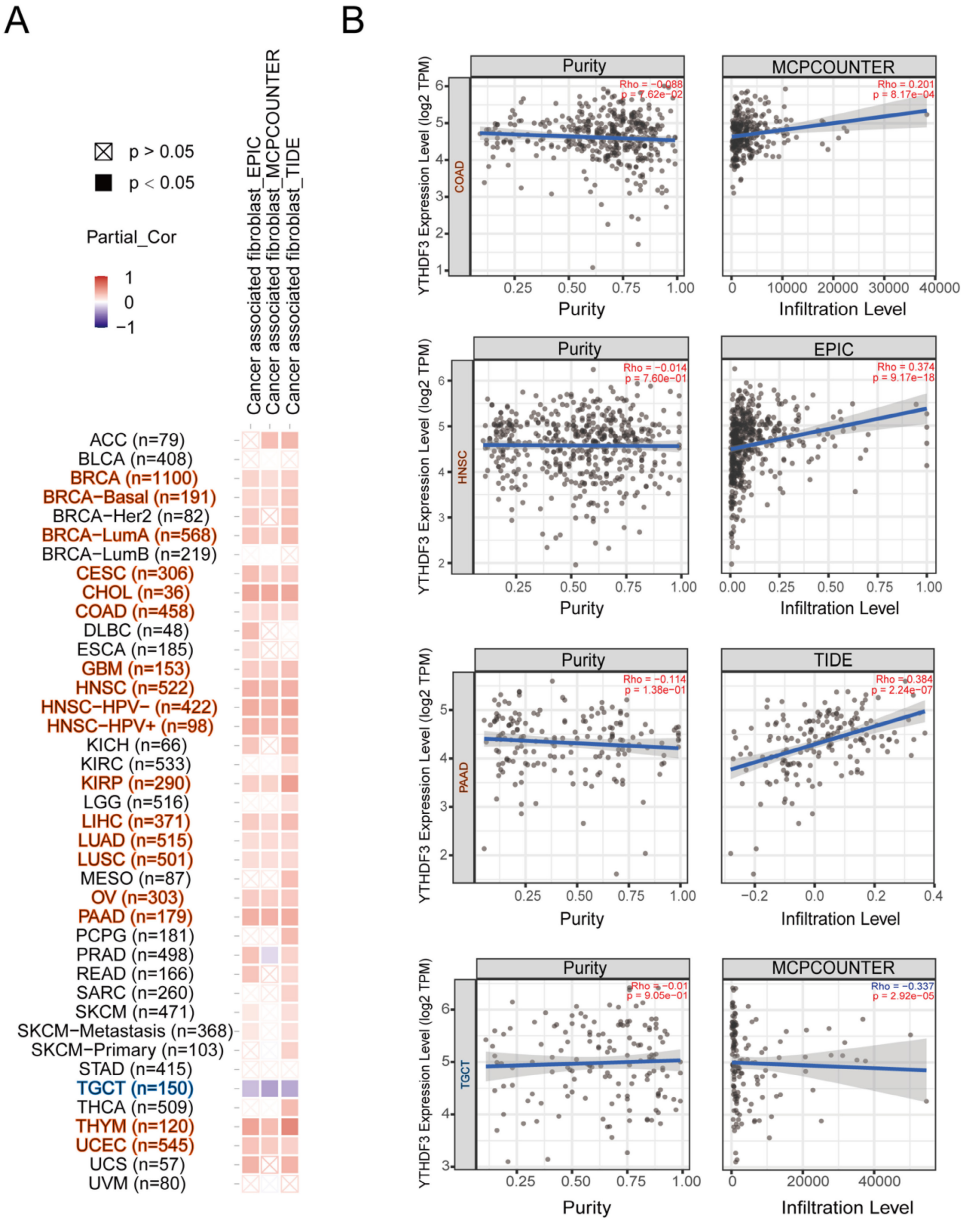
** Correlation analysis between YTHDF3 expression levels and immune infiltration in pan-cancer.** (A) The relationship between YTHDF3 expression levels in pan-cancer and cancer associated fibroblasts were compared though “TIDE”, “MCPCOUNTER” and “EPIC” databases. (B) Expression levels of *YTHDF3* mRNA expression in many cancers (COAD, HNSC, PAAD, and TGCT) were related to the immune infiltration of cancer associated fibroblasts. Significance is indicated by *P* < 0.05.

Tumor-infiltrating immune cells play a critical role in the current tumor treatment process, and these cells are closely related to the occurrence, development, and metastasis of tumors. We first explored the relevance of YTHDF3 in pan-cancer and immune cells. As shown in **Figure S7A-F**, we found that YTHDF3 expression was negatively correlated with T cells in most cancer types; thus, we decided to conduct the study shown in **Figure 6A-B**. It is well known that cancer-associated fibroblasts are involved in regulating the functions of various tumor-infiltrating immune cells. Therefore, we searched other databases for cancer-associated fibroblasts for further research. Here, we show the potential relationship between cancer-associated fibroblasts and YTHDF3 gene expression in the “TIDE”, “MCPCOUNTER”, and “EPIC” databases. After a series of analyses, we observed a statistically significant positive correlation between immune immersion in cancer-associated fibroblasts and YTHDF3 expression in BRCA (n = 1100), BRCA-basal (n = 191), BRCA-LumA (n = 568), CESC (n = 306), CHOL (n = 36), COAD (n = 458), GBM (n = 153), HNSC (n = 522), HNSC-HPV- (n = 422), HNSC-HPV+ (n = 98), KIRP (n = 290), LIHC (n = 371), LUAD (n = 515), LUSC (n = 501), OV (n = 303), PAAD (n = 179), THYM (n = 120), and UCEC (n = 545) by combining the results from the three databases (**Figure 6A**). Notably, there was a negative correlation between immune immersion in cancer-associated fibroblasts and YTHDF3 expression in TGCTs (n = 150). Scatter plot data of the abovementioned tumors are shown in **Figure 6B**. For example, according to the MCPCOUMTER algorithm, YTHDF3 expression levels in COAD are positively correlated with the level of immune infiltration in cancer-associated fibroblasts (COR = 0.201; *P* = 8.17 x 10^-4^), as depicted in **Figure 6B**. These results suggest that YTHDF3 expression in different cancer types is closely related to an immunosuppressive microenvironment.

### YTHDF3 is highly expressed in most cancer tissues and localized in the cytoplasm in cancer cells

**Figure 7:**
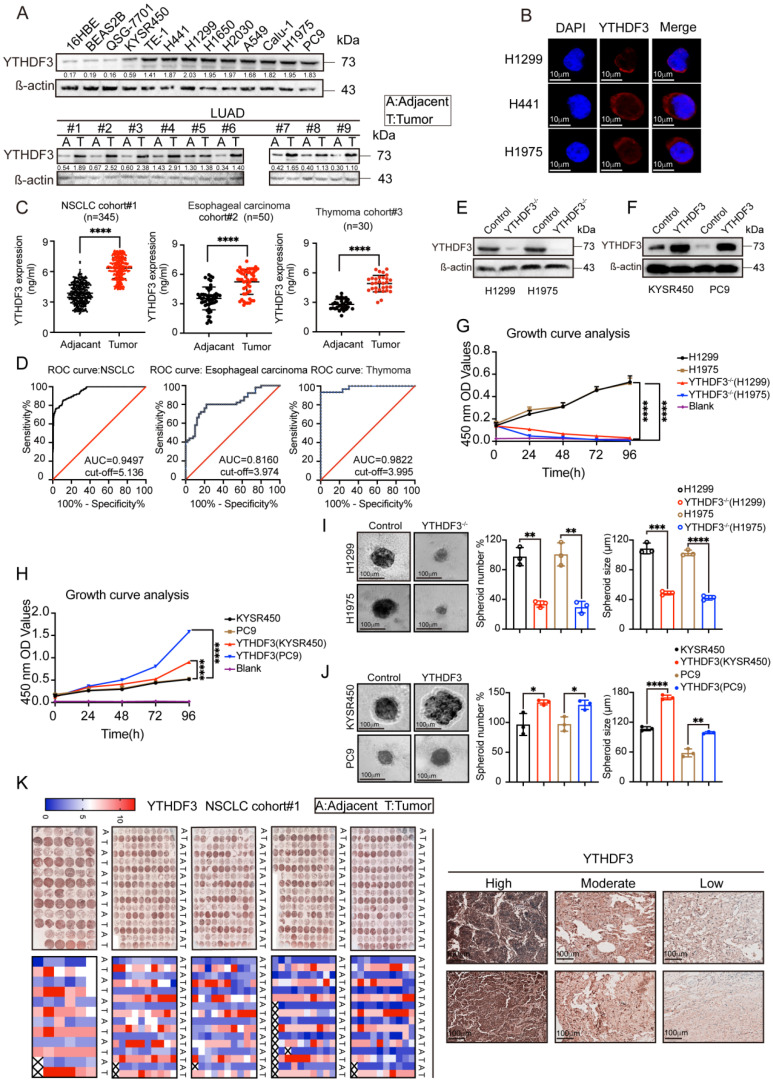
**YTHDF3 is upregulated in pan-cancer and correlated with poor clinical outcome.** (A) The intracellular expression levels of YTHDF3 protein in pan-cancer cells and normal cells, as measured by western blotting (WB). Paired human normal (N) and LUAD tumor (T) tissues were analyzed by WB from #1 to #9. (B) YTHDF3 was stained by immunofluorescence (IF) verifying its localization in H1299, H441 and H1975 cells. Scale bar 10 μm. (C) The YTHDF3 protein expression levels in paired adjacent-tumor tissues from NSCLC cohort #1 (n = 345), esophageal carcinoma cohort #2 (n = 50) and thymoma cohort #3 (n = 30), as measured by ELISA. (D) Receiver operating characteristic (ROC) curves and the areas under the ROC curve (AUC) values were plotted and calculated. (E-F) The WB assay confirmed that the knockout efficiency of YTHDF3 in H1299 and H1975 cells, and overexpression efficiency of YTHDF3 in KYSR450 and PC9 cells. (G-H) MTT assays were used to measure the proliferation rates of tumor cells in the indicated groups. (I) 3D tumor-sphere formation assays of control and YTHDF3-knockout H1299 and H1975 cells. Scale bar 100 μm. (J) 3D tumor-sphere formation assays of control and YTHDF3-overexpressing KYSR450 and HepG2 cells. The relative spheroid number and spheroid size were also quantified with histograms (I-J). (K) Immunohistochemistry (IHC) of NSCLC tissue microarray (TMA). The TMA-IHC images and IHC score of YTHDF3 in paired adjacent-tumor tissues from cohort #1 are presented. The representative high, moderate, and low expression of YTHDF3 images are also displayed in right panel. Data are presented as mean ± standard error of the mean (SEM) from at least three independent experiments. Statistical analysis was performed using a t-test (C, I, J) or two-way ANOVA (G, H). *P < 0.05, **P < 0.01, ***P < 0.001, and ****P < 0.0001.

We examined YTHDF3 protein expression levels in 13 different cell lines, including normal and cancer cells, by WB analysis. We then detected YTHDF3 expression in cancer tissue and adjacent tissue samples from nine lung adenocarcinoma patients. As shown in **Figure 7A**, the WB analysis suggested that YTHDF3 is highly expressed in most cancer cell lines. YTHDF3 expression was also found to be upregulated in lung adenocarcinoma tissues compared with adjacent tissue samples. These results provide strong evidence suggesting that YTHDF3 is overexpressed in tumor tissues.

To further characterize the function of the m^6^A-modified RNA methyl reader protein YTHDF3 and determine its subcellular localization, we next performed immunofluorescence staining. The results showed strong diffuse YTHDF3 expression in the cytoplasm of cell lines (H1299, H441, and H1975) (**Figure 7B**), suggesting that YTHDF3 is expressed in the cytoplasm of cancer cells.

To further illustrate the expression of YTHDF3, we selected 345 pairs of lung adenocarcinoma patients, 50 pairs of esophageal cancer patients, and 30 pairs of thymoma patients to monitor the immune response in cancer tissues and adjacent tissues by ELISAs. Baseline characteristics for the unmatched and matched sample are presented in **Supplementary Table 1-6**. Based on these data, we plotted ROC curves. The results showed that the YTHDF3 expression levels were higher in tumor tissues compared with those in the adjacent tissues (**Figure 7C**). From the ROC curve analysis, the AUC values were 0.9497 for NSCLC, 0.8160 for esophageal carcinoma, and 0.9822 for thymoma (**Figure 7D**). We also investigated the clinical relevance of YTHDF3 in cohort #1 (n = 345). By immunohistochemistry, we found YTHDF3 to be upregulated in tumors compared with adjacent tissues (**Figure 7K**). These conclusions indicate that YTHDF3 is abnormally expressed in cancer tissues and has potential diagnostic value.

### Overexpression and downregulation of YTHDF3 are connected to proliferative phenotypes

To determine the role of YTHDF3 in the proliferative phenotype in cells, we used the CRISPR/Cas9 system to generate YTHDF3 knockout H1299 and H1975 cell lines. Subsequently, YTHDF3 was overexpressed in KYSR450 and PC9 cells by a lentiviral-based YTHDF3 overexpression method (see Methods for details) (**Figure 7E-F**). Using knockout and overexpression cell models, we individually examined cell proliferation in the previously mentioned cell lines using the MTT method. Moreover, 3D spheroids are more likely to mimic tumors *in vivo*. To further confirm the role of YTHDF3 in cell proliferation, we also employed 3D tumor sphere formation assay experiments to re-evaluate the function of YTHDF3 in spheroid formation in several cell lines. The results of the MTT and 3D tumor sphere formation assay experiments showed that the proliferation abilities of H1299 and H1975 cells were reduced with YTHDF3 knocked out, while overexpressing YTHDF3 increased the proliferation of KYSR450 and PC9 cells (**Figure 7G-J**). Taken together, these data validate the pro-proliferative phenotype of YTHDF3. Furthermore, these data suggest that YTHDF3 is involved in cancer cells proliferation and that YTHDF3-high cells may have a proliferative advantage.

## Discussion

As members of the YTH protein family, YTHDF1, YTHDF2 and YTHDF3 have characterized roles in the cytoplasm and nucleus 31. Early reports on YTHDF family proteins have focused on YTHDF1 and YTHDF2 32. Currently, the prevailing view is that YTHDF3 and other YTHDFs function in an integrated and collaborative manner to affect the fundamental biological processes related to m^6^A 27. Previous studies have shown that YTHDF3 plays an important role in the occurrence and development in many types of cancer 33. In addition, several studies have shown that the m^6^A adjustment factor YTHDF3 is important in breast and colorectal cancers 5, 34. Although the current study provides an overall picture of YTHDF3 in some cancers, one limitation of this work is that a detailed analysis of YTHDF3 in pan-cancer is lacking. We aimed to analyze the role of YTHDF3 from a pan-cancer perspective. In the first step of our research, we used the TCGA database to determine the YTHDF3 expression levels in cancer and normal tissues. The results showed that YTHDF3 expression in tumors, from most cancer types other than ACC, CESC, DLBC, KIRC, KIRP, OV, and PCPG had a significantly higher expression, which was consistent with previous breast cancer and rectal cancer studies 34, 35. It was recently reported that YTHDF3 expression was associated with ocular melanoma and poor patient outcomes 36. Therefore, we speculated that high expression of YTHDF3 in most cancers has prognostic significance. In support of this conjecture, we investigated the relationship between YTHDF3 expression and prognosis. Patients with high expression levels of YTHDF3 had poor prognoses in BRCA, CESC, LGG, UVM, KICH, PAAD, SARC, OV, GBMLGG, UCEC, LIHC, THCA, and DLBC. In addition, we observed that *YTHDF3* mRNA levels was upregulated in breast cancer. Furthermore, our study found that YTHDF3 expression is related to various disease stages and patient ages, prompting us to use TCGA database to draw ROC curves and assess the diagnostic value of YTHDF3 in these cancer types. Herein, we demonstrated that YTHDF3 is a potential prognostic pan-cancer biomarker using diverse bioinformatic tools.

Most human cancers, contain many different types of mutations, most of which are missense mutations that significantly increase the risk of certain cancers. YTHDF3 gene amplification occurs frequently and leads to high expression levels in human breast cancer 33. Our results are consistent with these previously reported findings. Moreover, YTHDF3 was frequently amplified (6%) and contributed to the high expression levels of YTHDF3 in human breast cancer. In addition, breast cancer patients with changes in YTHDF3 expression have worse OS rates compared with those with no YTHDF3 change 33, which is consistent with our findings. Therefore, YTHDF3 may be considered a promising biomarker for predicting breast cancer patient survival. Gene copy number is also an important consideration, but often neglected phenomenon. CNVs are important factors leading to the abnormal upregulation of oncogenes. A key role for CNV in disease and evolution has been demonstrated by Eichler et al. 37. A recent study showed that the YTHDF3 copy number increase in LIHC was significantly higher than in other cancers 33, where it was amplified by more than 6% 38, suggesting that YTHDF3 may be an important oncogene that is selected during cancer evolution.

An increasing number of novel anticancer agents have been developed 39. In recent years, researchers have begun targeting checkpoint receptors for anticancer immunotherapy 40. The specific details regarding YTHDF3 as a potential novel target for immunotherapy should be investigated further. Interestingly, we observed a statistically significant negative correlation between YTHDF3 expression levels and the degree of T-cell immune infiltration in many types of cancer. In addition, our findings are the first to suggest that a relationship exists between YTHDF3 expression and immune invasion levels of cancer-associated fibroblasts across cancers. The results show that YTHDF3 expression is significantly different in various immune subtypes in most types of cancer, which could demonstrate that YTHDF3 is a promising cancer diagnostic biomarker that participates in immune regulation. The correlation between YTHDF3, MSI, and the TMB also suggested that YTHDF3 is closely related to the TME across cancers. In summary, the above results indicate the potential of YTHDF3 as a target in anticancer immunotherapy.

Finally, we also used the existing tissue and cell samples to examine YTHDF3 expression from various perspectives. We performed gene overexpression and knockout experiments and observed that YTHDF3 has an effect on the proliferation phenotype.

Taken together, these results provide strong evidence. However, although we performed a comprehensive systematic analysis of YTHDF3 expression, there are still some limitations to this study. First, the microarray and sequencing data from different databases suggest differences in size and specificity, which could lead to deviations in the system. Notably, from the ROC curve analysis, the AUC values were as follows: NSCLC: AUC = 0.9497, esophageal cancer: AUC = 0.8160, and thymoma: AUC = 0.9822 (Fig. 7D). We found that the AUC value being 0.9822 for thymoma may be because there were fewer samples for this cancer. Additional experiments are necessary to demonstrate the potential function of YTHDF3 in cancer, which can increase the credibility of our results. Overall, we have shown in this study that YTHDF3 expression, immune cell infiltration, and prognosis are closely related in human cancers.

## Supplementary Material

Supplementary figures and tables.

## Abbreviations

YTHDF3: YTH N6-methyladenosine RNA binding protein 3
MSI: microsatellite instability
TMB: tumour mutation burden
TPM: transcripts per million
FPKM: fragments per kilobase of exon model per million mapped fragments
CNA: copy number alteration
SNV: single nucleotide variation
CNV: copy number variation
TCGA: The Cancer Genome Atlas
GTEx: Genotype-Tissue Expression
CPTAC: Clinical Proteomic Tumor Analysis Consortium
TCGA-ACC: Adrenocortical carcinoma
TCGA-BLCA: Bladder Urothelial Carcinoma
TCGA-BRCA: Breast invasive carcinoma
TCGA-CESC: Cervical squamous cell carcinoma and endocervical adenocarcinoma
TCGA-CHOL: Cholangiocarcinoma
TCGA-COAD: Colon adenocarcinoma
TCGA-COADREAD: Colon adenocarcinoma/Rectum adenocarcinoma Esophageal Carcinoma
TCGA-DLBC: Lymphoid Neoplasm Diffuse Large B-cell Lymphoma
TCGA-ESCA: Esophageal carcinoma
TCGA-FPPP: FFPE Pilot Phase II
TCGA-GBM: Glioblastoma multiforme
TCGA-GBMLGG: Glioma
TCGA-HNSC: Head and Neck squamous cell carcinoma
TCGA-KICH: Kidney Chromophobe
TCGA-KIPAN: Pan-kidney cohort (KICH+KIRC+KIRP)
TCGA-KIRC: Kidney renal clear cell carcinoma
TCGA-KIRP: Kidney renal papillary cell carcinoma
TCGA-LAML: Acute Myeloid Leukemia
TCGA-LGG Brain: Lower Grade Glioma
TCGA-LIHC: Liver hepatocellular carcinoma
TCGA-LUAD: Lung adenocarcinoma
TCGA-LUSC: Lung squamous cell carcinoma
TCGA-MESO: Mesothelioma
TCGA-OV: Ovarian serous cystadenocarcinoma
TCGA-PAAD: Pancreatic adenocarcinoma
TCGA-PCPG: Pheochromocytoma and Paraganglioma
TCGA-PRAD: Prostate adenocarcinoma
TCGA-READ: Rectum adenocarcinoma
TCGA-SARC: Sarcoma
TCGA-STAD: Stomach adenocarcinoma
TCGA-SKCM: Skin Cutaneous Melanoma
TCGA-STES: Stomach and Esophageal carcinoma
TCGA-TGCT: Testicular Germ Cell Tumors
TCGA-THCA: Thyroid carcinoma
TCGA-THYM: Thymoma
TCGA-UCEC: Uterine Corpus Endometrial Carcinoma
TCGA-UCS: Uterine Carcinosarcoma
TCGA-UVM: Uveal Melanoma
TARGET-OS: Osteosarcoma
TARGET-ALL: Acute Lymphoblastic Leukemia
TARGET-NB: Neuroblastoma
TARGET-WT: High-Risk Wilms Tumor
