# Supplementary material for: Integrated Clinical and Prognostic Analysis of the m6A RNA Methylation Regulator YTHDF3 in Pan-Cancer and its Correlation with Cancer Cell Proliferation
Source: J Cancer. 2022 Nov 21;13(15):3623–39. doi: 10.7150/jca.78403 (PMC9809312; doi:10.7150/jca.78403)
Supplement: Supplementary file 1 — Supplementary figures and tables. [file jcav13p3623s1.pdf]

Supplementary Tables

Table 1. Comparison of confounding factors in different NSCLC stages before and after PSM

Clinicopathologic	Stage (before PSM)			Stage (after PSM)		
Features	I-II	III	*P*	I-II	III	*P*
	n=230	n=115		n=93	n=93	
Ages			0.305			0.213
≥65	108	61		40	40	
<65	122	54		53	53	
Gender			0.208			0.097
Male	116	67		55	55	
Female	114	48		38	38	
Smoking			0.745			1.000
Yes	108	50		46	46	
No	122	65		47	47	

Table 2. The correlation of YTHDF3 expression with clinicopathologic features of NSCLC before and after PSM

Clinicopathologic Features	YTHDF3 (before PSM)			YTHDF3 (after PSM)		
	Low	High	*P*	low	high	*P*
Stage			0.000			0.000
I-II	69	161		20	73	
III	12	103		3	90	

Table 3. Comparison of confounding factors in different stages of Esophageal carcinoma before and after PSM

Clinicopathologic	Stage (before PSM)			Stage (after PSM)		
Features	I-II	III	*P*	I-II	III	*P*
	n=35	n=15		n=15	n=15	
Ages			0.888			1.000
≥65	17	7		7	7	
<65	18	8		8	8	
Gender			0.852			0.607
Male	20	9		9	9	
Female	15	6		6	6	
Smoking			0.998			1.000
Yes	16	7		7	7	
No	19	8		8	8	

Table 4. The correlation of YTHDF3 expression with clinicopathologic features of Esophageal carcinoma before and after PSM

Clinicopathologic Features	YTHDF3 (before PSM)			YTHDF3 (after PSM)		
	Low	High	*P*	low	high	*P*
Stage			0.000			0.000
I-II	9	26		2	13	
III	1	14		1	14	

Table 5. Comparison of confounding factors in different stages of Thymoma before and after PSM

Clinicopathologic Features	Stage (before PSM)			Stage (after PSM)		
	I-II n=19	III n=11	*P*	I-II n=11	III n=10	*P*
Ages			0.146			0.227
≥65	10	3		3	3	
<65	9	8		8	7	
Gender			1.000			0.791
Male	11	7		3	6	
Female	8	4		8	4	
Smoking			1.000			0.754
Yes	12	7		7	6	
No	7	4		4	4	

Table 6. The correlation of YTHDF3 expression with clinicopathologic features of Thymoma before and after PSM

Clinicopathologic Features	YTHDF3 (before PSM)			YTHDF3 (after PSM)		
	Low	High	*P*	low	high	*P*
Stage			0.002			0.000
I-II	6	13		1	10	
III	1	10		1	9	

Supplementary Figures and Figure legends

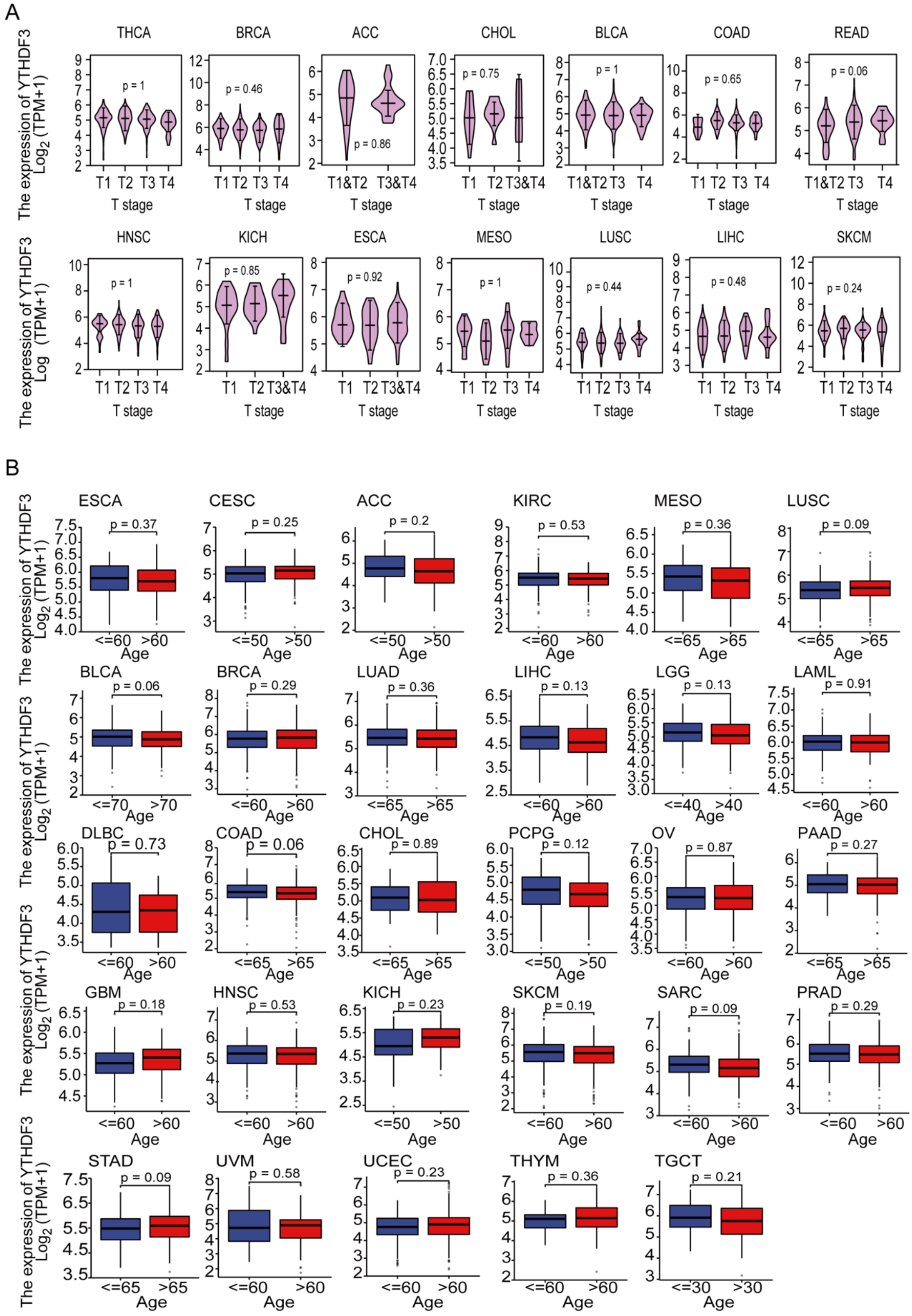

Figure S1. Clinical variables analysis of YTHDF3 in pan-cancer. (A) YTHDF3 expression level were regulated with clinical variables-stage (THCA, BRCA, ACC, CHOL, BLCA, READ, HNSC, KICH, ESCA, MESO, LUSC, COAD, LIHC, SKCM)

in TCGA human-cancer database; (B) The expression level of YTHDF3 gene were analyzed with clinical variables-Age (ESCA, CESC, ACC, KIRC, MESO, LUSC, BLCA, BRCA, LUAD, LIHC, LGG, LAML, DLBC, COAD, CHOL, PCPG, OV, PAAD, GBM, HNSC, KICH, SKCM, SARC, PRAD, STAD, UVM, UCEC, THYM, TGCT).

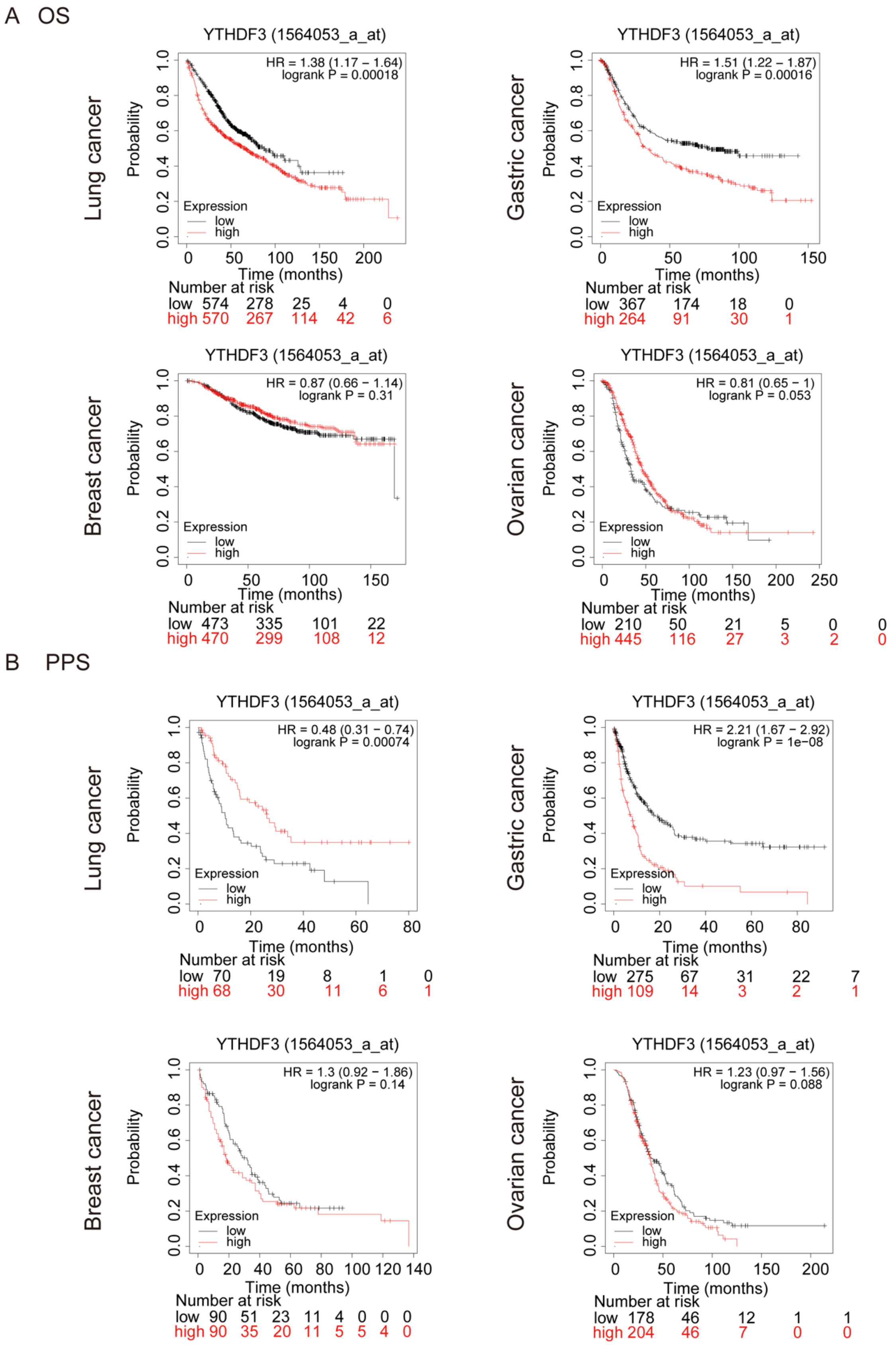

Figure S2. Kaplan-Meier survival curve based on YTHDF3 expression levels were analyzed in the Kaplan-Meier plotter database. (A) Comparison of different

expression levels of YTHDF3 gene in TCGA human cancer database from overall survival (Ovarian cancer, Lung cancer, Gastric cancer, Breast cancer). (B) Comparison of different expression levels of YTHDF3 gene in TCGA human cancer database from post-progression survival (Ovarian cancer, Lung cancer, Gastric cancer, Breast cancer).

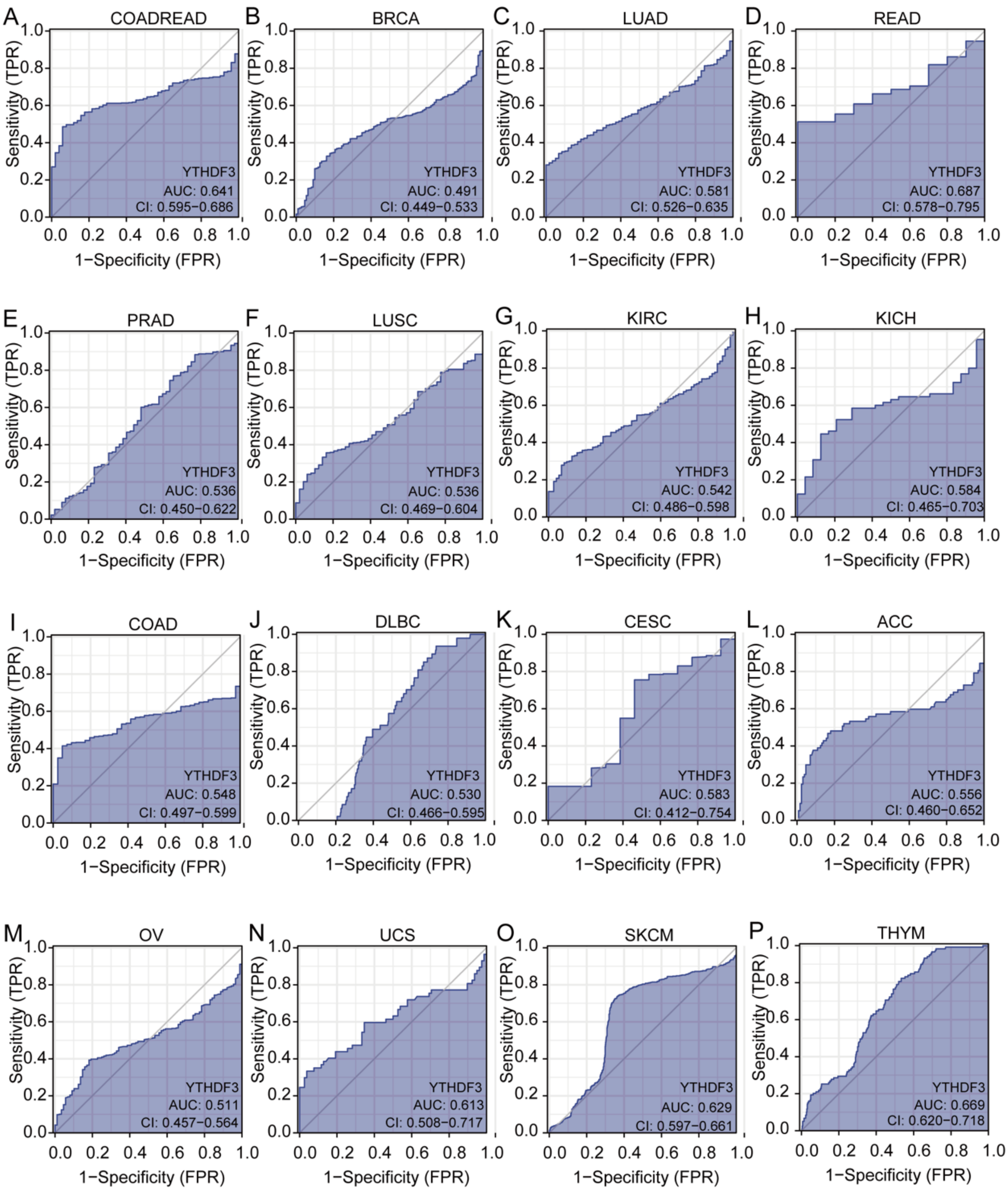

Figure S3. ROC curve was established to estimate the value of *YTHDF3* mRNA expression as a biomarker in pan-cancer. (A-P: COADREAD, BRCA, LUAD, READ, PRAD, LUSC, KIRC, KICH, COAD, DLBC, CESC, ACC, OV, UCS, SKCM, THYM).

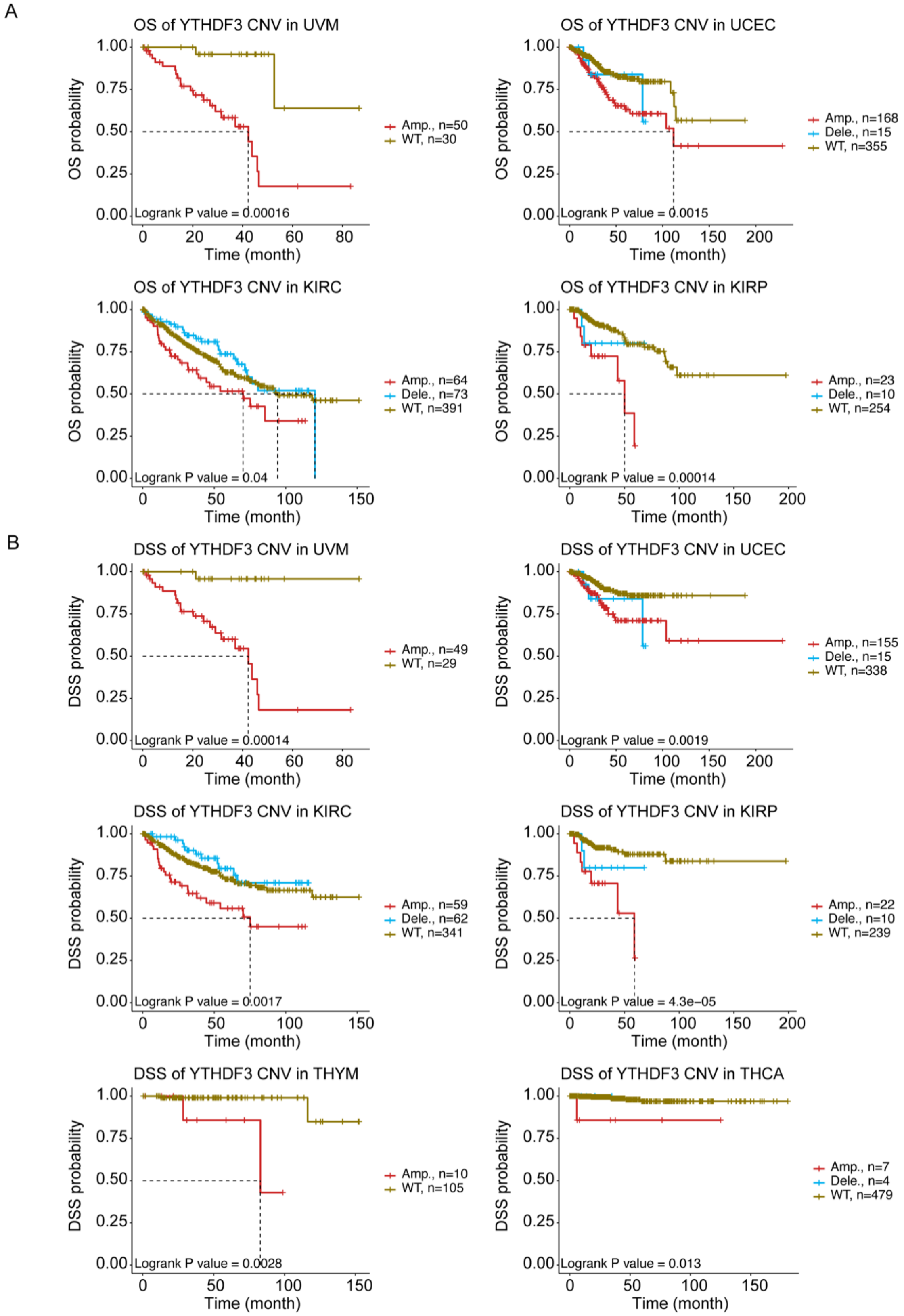

Figure S4. YTHDF3 associated with mutation were analyzed in the TCGA data source. (A) YTHDF3 CNV was linked to poor prognosis of overall survival (OS) for cancers of UVM, UCEC, KIRC and KIRP. (B) YTHDF3 CNV was linked to poor

prognosis of disease special survival (DSS) for cancers of UVM, UCEC, KIRC and KIRP, THYM and THCA.

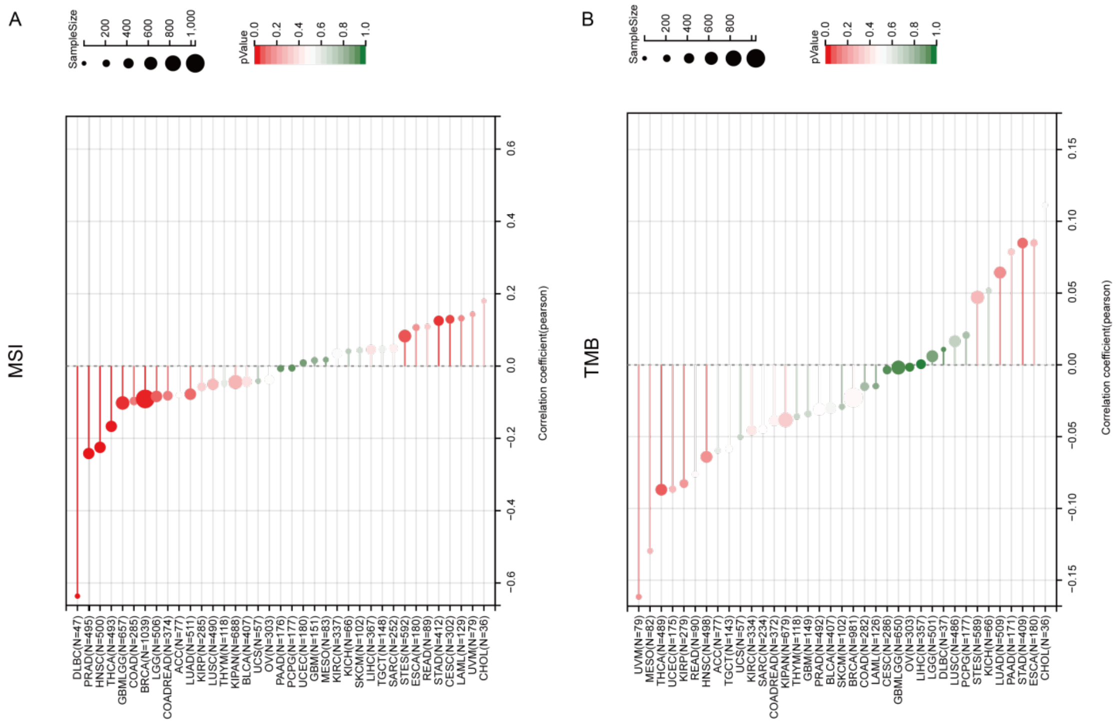

Figure S5. (A) The relationship between YTHDF3 expression and microsatellite instability (MSI) in pan-cancer. (B) The relationship between YTHDF3 expression and tumor mutational burden (TMB) in pan-cancer.

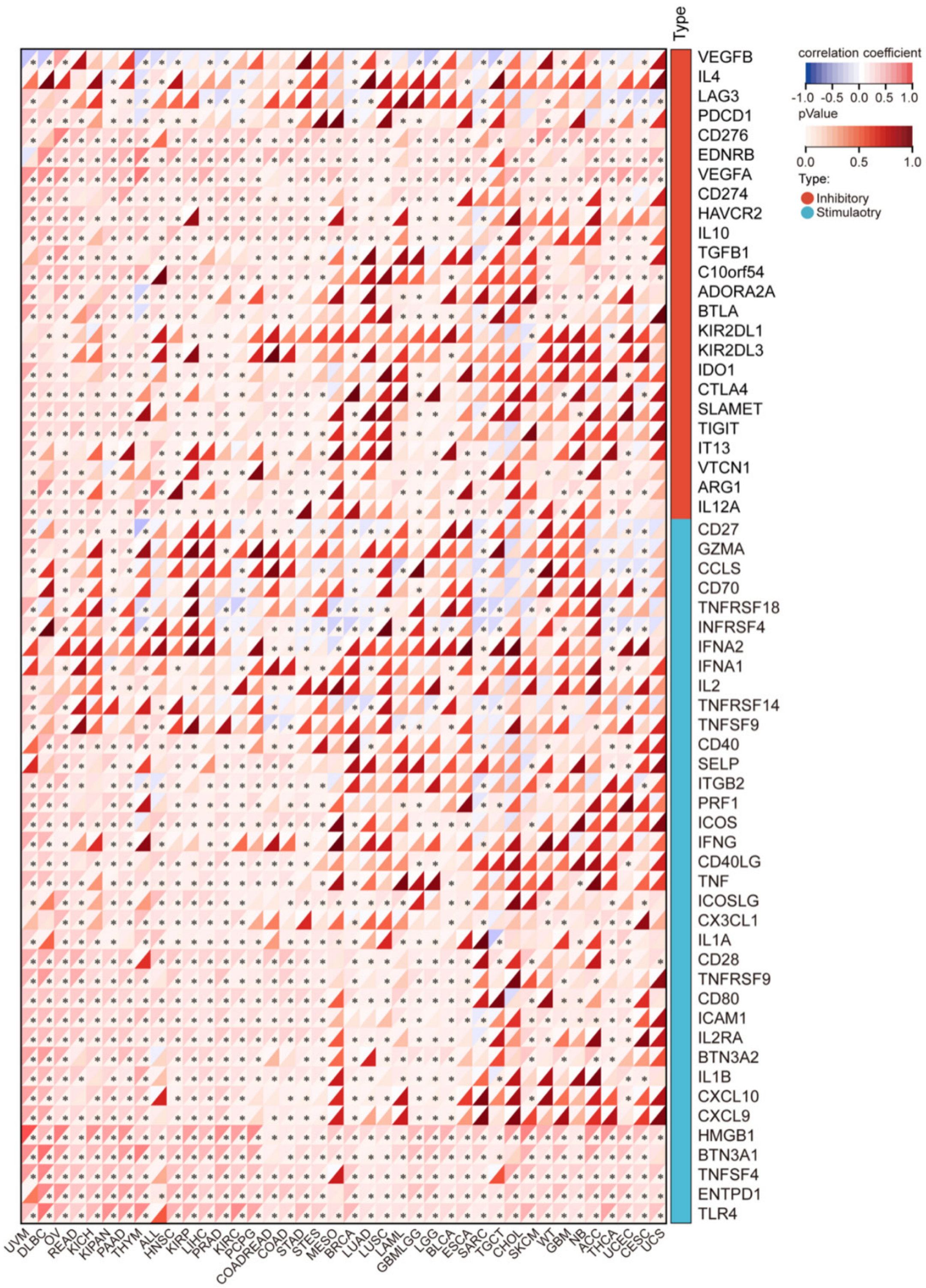

Figure S6. The relationship between YTHDF3 expression and pan-cancer immune checkpoint genes.

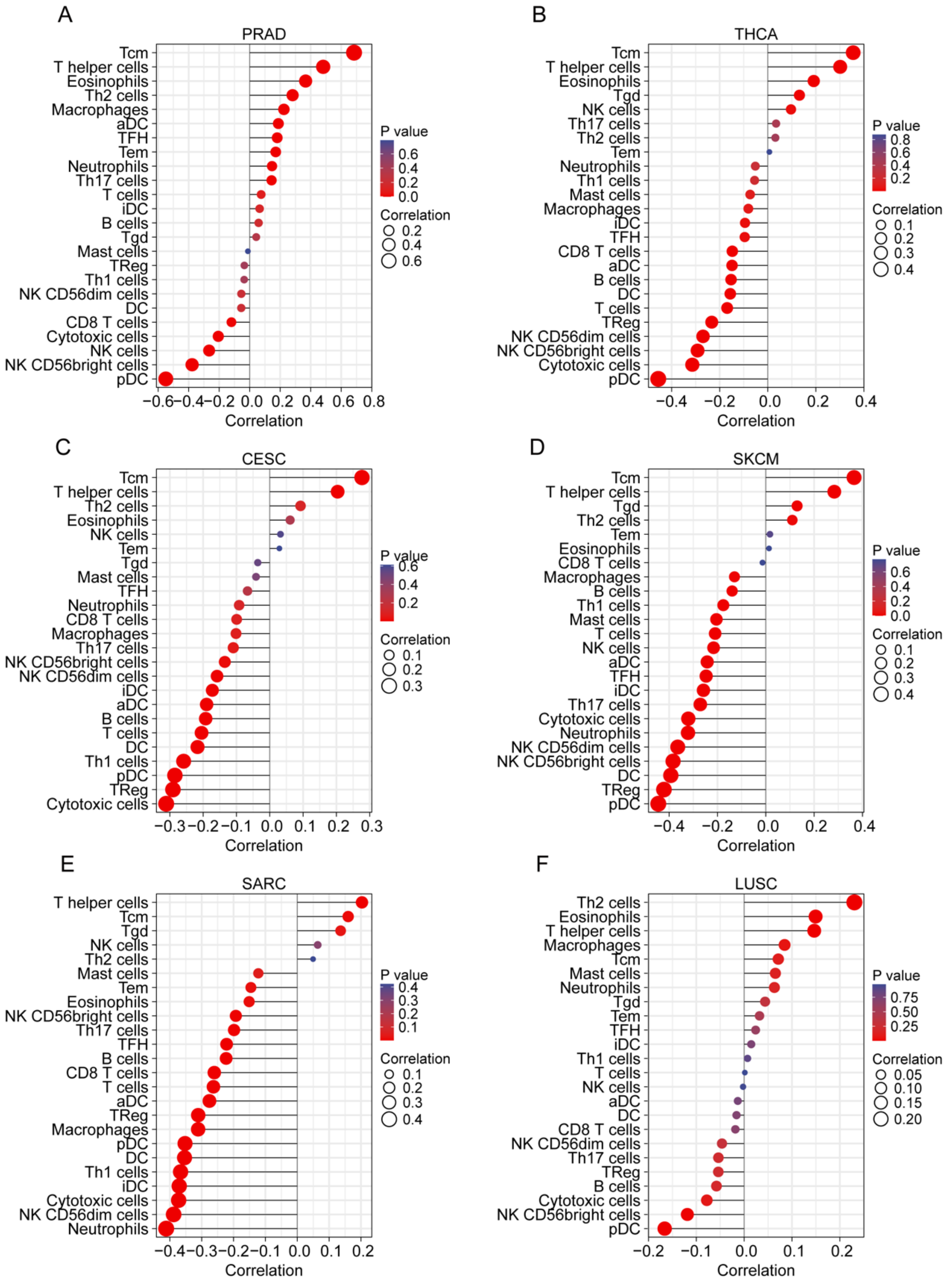

Figure S7. The correlation between YTHDF3 and immune cells was extracted from the pan-cancer TCGA data source. Results are displayed with Lollipop Map (A: PRAD, B: THCA, C: CESC, D: SKCM, E: SARC, F: LUSC).
